# Association of marital status and access to dental care among the Japanese population: a cross-sectional study

**DOI:** 10.1186/s12903-022-02311-1

**Published:** 2022-07-07

**Authors:** Yuko Inoue, Takashi Zaitsu, Akiko Oshiro, Miho Ishimaru, Kento Taira, Hideto Takahashi, Jun Aida, Nanako Tamiya

**Affiliations:** 1 Department of Oral Health Promotion, Graduate School of Medical and Dental Sciences, Tokyo Medical and Dental University, 1-5-45 Yushima, Bunkyo-ku, Tokyo 113-8510 Japan; 2 Department of Health Services Research, Faculty of Medicine, Institutes of Medicine, University of Tsukuba, Building #861, 1-1-1 Tenno-dai, Tsukuba, Ibaraki 305-8575 Japan; 3 National Institute of Public Health, 2-3-6 Minami, Wako-shi, Saitama 351-0197 Japan

**Keywords:** Oral symptoms, Oral health, Dental treatment, Dental attendance, Marital status

## Abstract

**Background:**

Health disparities according to marital status have been reported worldwide. Although spouses provide an important social network that influences heath behaviors, limited studies have examined the association between marital status and access to dental care. Therefore, this study aimed to analyze the association between marital status and access to dental care.

**Methods:**

A secondary analysis of the 2013 Comprehensive Survey of Living Conditions in Japan which is a national survey, was performed in this study. Out of 367,766 respondents, 4111 respondents, aged over 40 years who selected oral symptoms as their most concerning subjective symptom were recruited as participants. The independent variable of interest was marital status—married or non-married (single, divorced, widowed); and the dependent variable was access to dental care. We performed Poisson regression analyses stratified by sex with adjustment for age, educational status, employment, equivalent household expenditure, and smoking habits.

**Results:**

Among respondents who reported oral symptoms, 3024 were married, and 1087 were non-married. Further, 29.4% and 40.4% of married and non-married men, respectively, did not receive dental treatment for their symptoms. Meanwhile, 27.5% and 25.0% of married and non-married women, respectively, did not receive dental treatment for their symptoms. The prevalence ratio for not receiving dental treatment was significantly higher among non-married men (prevalence ratio: 1.33; 95% confidence interval: 1.14–1.56) than among married men. However, no significant association was observed among women.

**Conclusions:**

Non-married men were highly unlikely to receive dental treatment than married men, while no significant association was observed among women. The results implicate the importance of implementing a public dental health policy for protecting the dental health of non-married individuals.

## Background

Social networks affect healthcare behaviors as a social determinant of health [1, 2]. Spouses generally provide the closest social network; therefore, marital status is considered to have considerable effects on health behaviors. Marital status influences health behaviors [3], lifestyle, and health status [4, 5]. It is also associated with cancer screening behavior [6] and medical care access [7, 8]. Consequently, single individuals have a higher mortality rate than married individuals [9]. An association between marital status and tooth loss has been reported in dental health [10]. However, only a few studies have examined the association between marital status and access to dental care [11–13].

Oral diseases are one of the most prevalent diseases [14]. However, in many countries, access to dental care is often difficult because coverage of dental care is limited in the context of universal health coverage [15, 16]. Oral diseases are highly prevalent and affect approximately 3.5 billion people worldwide [14]; untreated dental caries, in particular, is the most common oral disease, affecting 34.1% of the world population [17]and about 30% of Japanese adults [18]. However, many people with oral diseases especially people with a low income and older adults experience difficulty in accessing dental care cannot visit dental clinics[19].

There are several social determinants of access to dental care. Income-based inequalities in access to dental care have been observed in many countries [11, 20]. This is also the case for Japan [21], which has one of the lowest out-of-pocket dental expenditures among the Organization for Economic Co-operation and Development (OECD) countries because of the extensive coverage of dental care by public universal care insurance [22]. Other types of social determinants, such as education [16, 23] and employment status [24], have also been reported. However, most studies on access to dental care have focused on socioeconomic status as a social determinant. Therefore, this study examined the association between marital status and access to dental care among individuals with oral symptoms in Japan.

## Methods

### Study sample

**Fig. 1:**
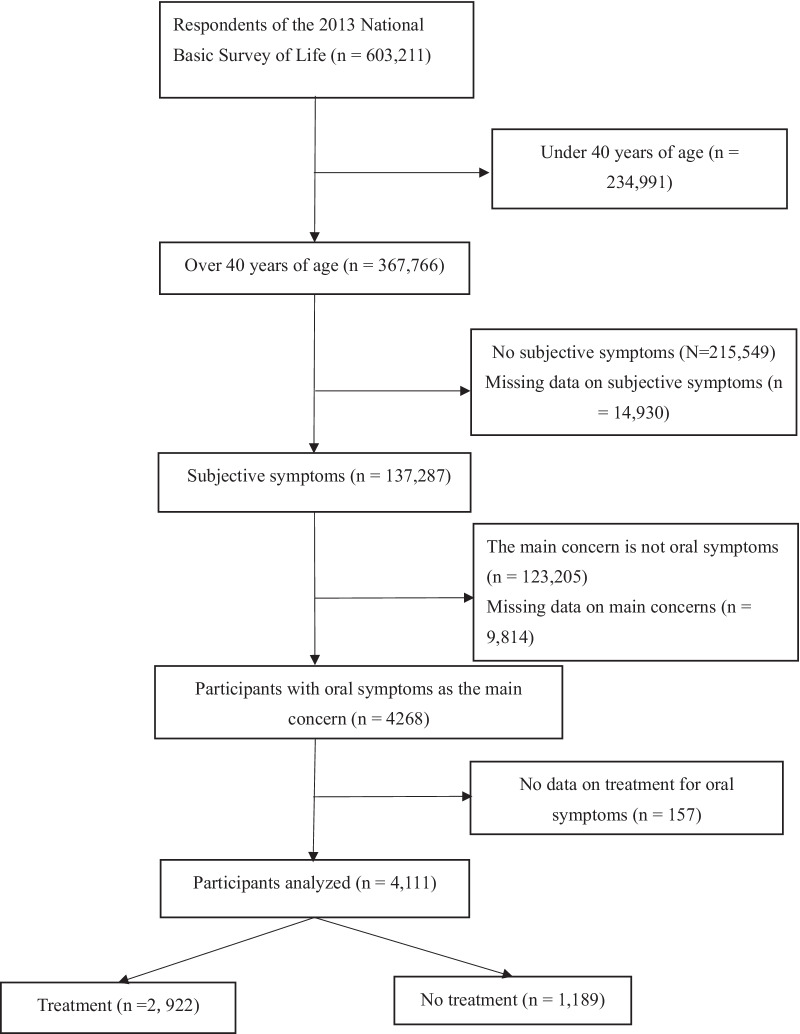
Flow chart of participant enrollment

This cross-sectional study was based on a secondary analysis of the data obtained from the 2013 Comprehensive Survey of Living Conditions, a cross-sectional and nationally representative survey conducted by the Japanese Ministry of Health, Labour and Welfare (MHLW) [25]. The survey was conducted in 5,530 districts randomly selected from approximately 1 million districts across the country and included all residents living in those districts (approximately 300,000 households, or 740,000 people). The response rate to the survey was 78.4% (234,383/295,367 households). We identified the analyzed participants with dental symptoms from among the survey participants based on their answers to self-reported questions in the health questionnaire. Further, data were analyzed only from people aged 40 years or older because the prevalence of periodontal disease increases from around this age [26, 27]. Excluding missing data on subjective symptoms, a total of 4111 subjects were finally included in the analysis (Fig. 1).

Approval was obtained from the MHLW to use the data according to Article 36 of the Statistical Act; the MHLW followed the prescribed protocol for the provision of anonymized data. Participants were waived from giving their consent because the National Consumer Survey is a government statistical survey and does not identify specific individuals.

### Dependent variable

The prevalence of receiving dental treatment for any oral symptoms was used as the dependent variable. The subjective symptom questionnaire included 42 items on whether respondents experienced any subjective symptoms of an illness or injury, including general symptoms, respiratory symptoms, or musculoskeletal symptoms, in the last few days. They were asked to select the one symptom that bothered them the most. Oral symptoms included toothache, gum symptoms, and chewing difficulties. Those who reported one of these oral symptoms as their most serious concern were selected for the analyzed population. Among them, those who visited dental clinics were classified as the “treatment group,” and those who did not visit dental clinics were classified as the “non-treatment group.”

### Independent variable

Marital status was classified as married and non-married (single, widowed, and divorced).

### Covariates

Age, employment status, educational level, equivalent household expenditure, and smoking status were used as covariates. The choices of questions with fewer responses were integrated. Age was divided into four groups: 40–49, 50–59, 60–69, over 70 years. Employment status was grouped as “employed” or “unemployed.” Educational status was categorized into four groups: “up to junior high school,” “high school,” “college,” and “university or higher degree.” While the survey data did not include annual income, data on monthly household expenditure were available. Therefore, equivalent household expenditure was calculated by dividing household expenditure by the square root of the number of household members. Equivalent household expenditure was divided into four categories: < 100,000, 100,000–149,999, 150,000–199,999, and ≥ 200,000 yen. Regarding smoking status, responses of “daily smoking” and “occasional smoking” were grouped as “current smoker,” whereas responses of “no smoking” and “no smoking for more than one month” were grouped as “non-smoker.”

### Statistical analysis

A chi-squared test was performed to analyze dental treatment for oral symptoms, for men and women separately. The association between marital status and access to dental care with adjustment for covariates was analyzed using Poisson regression analysis, because prevalence of non-dental treatment was high for logistic regression analysis [28]. Crude model and adjusted model were built with employment, education, smoking status, and equivalent household expenditure to identify risk factors for not receiving dental treatment stratified by sex. Poisson regression analysis was also performed in which the interaction term “sex × marital status” was added to the adjusted model to determine any sex difference in the association between marital status and access to dental care. For missing values, multiple imputation (MI) was used [29]. Each missing value was replaced with a substitute set of plausible values using MI with chained equations to create 10 complete datasets. The following variables were used to create the complete datasets; sex, age, marital status, employment status, education level, equivalent household expenditure, smoking status, and dental treatment. All data analyses were performed using STATA® 17 (Stata Corporation, College Station, TX, USA). Statistical significance was set at *P* < 0.05.

## Results

Overall, 73.6% of the respondents were married, and 26.4% were non-married. The percentage of married men (79.6%) was higher than that of married women (68.5%). Compared to married individuals, a higher proportion of non-married individuals were younger, unemployed, smokers, and had lower education. Male participants showed the same characteristics, but there was no difference in terms of employment status between married and non-married women.

Table 1 shows the characteristics of the treatment and non-treatment groups by sex. A total of 4111 people (1862 men and 2249 women) reported oral symptoms as their most serious concern. The overall percentage of individuals with untreated oral symptoms was 28.9%, which was higher in men than in women. Overall, compared to those who received dental treatment, those who did not receive dental treatment were likely to be younger, employed, highly educated, and current smokers. In both men and women, there were significant differences in dental treatment behavior by age, marital status, all socioeconomic characteristics, and smoking status (*p* < 0.01).

**Table 1:** Characteristics of participants with any oral symptoms as their most concerning subjective symptom (N = 4111)

	Overall		Men			Women		
			Treatment n = 1273 (68.4%)	No treatment n = 589 (31.6%)		Treatment n = 1649 (73.3%)	No treatment n = 600 (26.7%)	
	n	%	%	%	*P*	%	%	*P*
Age (years)								
40–49	754	18.3	67.4	32.6	< 0.01	64.8	35.2	< 0.01
50–59	966	23.5	63.1	36.9		70.0	30.1	
60–69	1270	30.9	67.5	32.5		77.7	22.3	
≥ 70	1121	27.3	75.4	24.6		76.6	23.4	
Marital status								
Married	3024	73.6	70.6	29.4	< 0.01	72.6	27.5	< 0.01
Non-married	1087	26.4	59.6	40.4		75.0	25.0	
Employment status								
Employed	2152	52.4	67.7	32.3	< 0.01	70.2	29.8	< 0.01
Unemployed	1959	47.6	69.6	30.4		75.6	24.4	
Education								
Up to junior high school	722	17.6	70.6	29.4	< 0.01	74.5	25.5	< 0.05
High school	2017	49.1	68.2	31.8		73.2	26.9	
College	670	16.3	64.0	36.0		72.2	27.8	
University or higher	702	17.1	69.0	31.0		74.6	25.4	
Equivalent household expenditure (yen)								
< 100,000	939	22.8	65.2	34.8	< 0.01	71.9	28.2	0.03
100,000–149,999	1392	33.9	70.2	29.8		74.2	25.8	
150,000–199,999	830	20.2	66.2	33.8		73.4	26.6	
≥ 200,000	950	23.1	70.9	29.1		73.4	26.6	
Smoking status								
Current smoker	896	21.8	62.7	37.3	< 0.01	67.3	32.7	< 0.01
Non-smoker	3216	78.2	71.5	28.5		74.0	26.0	

**Fig. 2:**
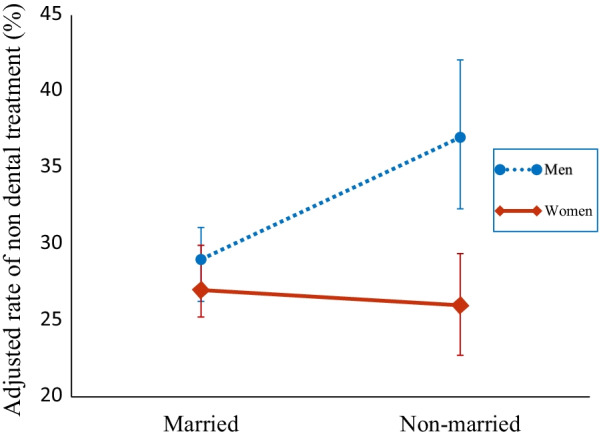
Interaction effects of sex and marital status on non-dental treatment (N = 4111)

Further, Table 2 shows that among men, the adjusted prevalence ratio for non-dental treatment was significantly higher in the non-married group at 1.33 (95% CI, 1.14–1.56) compared to the married group. In women, there was no significant association between marital status and dental treatment. Finally, in the model with the interaction term, there was a significant interaction of sex on the association of marital status with dental treatment (Fig. 2).

**Table 2:** Prevalence ratios for no dental treatment by Poisson regression analysis stratified by sex

Variable	Men (n = 1,862)			
	Crude		Adjusted	
	PR	95% CI	PR	95% CI
Marital status				
Married	1.00		1.00	
Non-married	1.37	1.14–1.65	1.33	1.14–1.56
Age (years)				
40–49	1.00		1.00	
50–59	1.13	0.94–1.37	1.22	1.00–1.48
60–69	1.00	0.83–1.20	1.09	0.89–1.34
≥ 70	0.76	0.61–0.94	0.84	0.64–1.09
Employment status				
Employed	1.00		1.00	
Unemployed	0.94	0.82–1.09	1.07	0.90–1.28
Education				
Up to junior high school	0.95	0.76–1.19	0.99	0.78–1.25
High school	1.03	0.87–1.22	1.00	0.84–1.19
College	1.16	0.91–1.48	1.11	0.87–1.42
University or higher	1.00		1.00	
Equivalent household expenditure (yen)				
< 100,000	1.19	0.98–1.46	1.19	0.97–1.45
100,000−149,999	1.02	0.85–1.24	1.03	0.85–1.25
150,000–199,999	1.16	0.94–1.43	1.20	0.98–1.48
≥ 200,000	1.00		1.00	1.00
Smoking status				
Current smoker	1.31	1.14–1.49	1.24	1.08–1.42
Non-smoker	1.00		1.00	

Variable	Women (n = 2249)			
	Crude		Adjusted	
	PR	95% CI	PR	95% CI
Marital status				
Married	1.00		1.00	
Non-married	0.91	0.76–1.09	0.93	0.79–1.08
Age (years)				
40–49	1.00		1.00	
50–59	0.85	0.71–1.03	0.85	0.70–1.03
60–69	0.63	0.52–0.77	0.64	0.52–0.79
≥ 70	0.67	0.55–0.81	0.67	0.53–0.85
Employment status				
Employed	1.00		1.00	
Unemployed	0.82	0.72–0.94	0.95	0.81–1.11
Education				
Up to junior high school	1.01	0.74–1.37	1.25	0.89–1.75
High school	1.06	0.79–1.42	1.16	0.86–1.57
College	1.10	0.81–1.49	1.10	0.81–1.49
University or higher	1.00		1.00	
Equivalent household expenditure (yen)				
< 100,000	1.06	0.87–1.29	1.05	0.86–1.28
100,000–149,999	0.97	0.81–1.17	0.95	0.79–1.14
150,000–199,999	1.00	0.81–1.23	0.99	0.80–1.22
≥ 200,000	1.00			
Smoking status				
Current smoker	1.26	1.03–1.53	1.13	0.92–1.38
Non-smoker	1.00		1.00	

*CI* confidence interval, *PR* prevalence ratio

## Discussion

This study examined the association between marital status and access to dental care for oral symptoms using data from the 2013 Comprehensive Survey of Living Conditions, a nationally representative sample of the Japanese population. The results indicate the association between marital status and access to dental care among those reporting oral symptoms, was observed only among men, not among women. Non-married men were more unlikely to receive dental treatment for their oral symptoms compared to married men.

This finding is consistent with previous studies examining the association between marital status and various health outcomes. For example, non-married men are at a higher risk of hypertension [30] and cardiovascular mortality [9, 31], psychiatric disorders [32, 33], and a lower rate of cancer screening and care [7, 34, 35] compared to married men. A German cohort study in the field of dentistry over 5 years reported that married individuals had a lower risk of tooth loss than non-married individuals [10]. Our study contributed to the knowledge about access to dental care and marital status.

Social network and influence are potential underlying mechanisms of the association between men’s marital status and dental visits. Married men possibly tend to be affected by the wife’s good dental behavior. Studies reported that health behaviors diffuse through the social network [36]. Women, in general, have better health awareness and health management skills [37]; therefore, their health behaviors toward dental treatment are also expected to be better, regardless of marital status. In fact, women are highly likely to undergo dental checkups than men [38], indicating that they are highly likely to be aware of their oral health. Thus, women’s good attitudes toward dental care are considered to influence their husbands’. The lack of this social influence from the wife would reduce dental visits among non-married men.

The current study results implicates the importance of the implementation of a public dental health policy for protecting the dental health of non-married individuals. According to a national census [39], the number of non-married people is increasing, and the rate of non-marriage for both men and women is expected to increase in the future due to increasing late marriages and divorce rates [40]. Including dental health care into general health policies is one possible measure to improve access to care among non-married individuals. For example, an intervention study conducted in the United Kingdom [41] reported health examinations that included dental checkups and assistance in arranging dental appointments for older adults and promoted dental visits. According to Japan’s Industrial Safety and Health Act [42], companies must provide medical examinations and guidance for workers, whereas dental examinations are mandatory only for workers who are exposed to toxic chemicals. Consequently, only a few companies offer dental checkups to their employees. In addition, while the government offers specific health checkups for people aged 40 years and older, dental checkups are not included in these health examinations. Including dental examination in these general health examination benefits could improve oral health.

The present study has some limitations and strengths. One is that it is a cross-sectional design, which precludes causal inferences about the association between marital status and dental care behavior. However, it is uncertain whether dental visits affect the likelihood of marriage; therefore, reverse causation due to the cross-sectional design may be unlikely. In addition, we could not consider several covariates influencing the present results. For example, several factors discourage people from receiving dental treatment, including geographical location [43], economic factors [23, 38, 44], time [45], and fear of receiving dental treatment [46]. The current study was unable to examine these factors; thus, further analysis is required. Additionally, from the questionnaire, we were only able to determine dental treatment among only those who answered that dental symptoms were their most concerning symptoms. Future research is needed to determine if consistent results are seen among those with milder dental symptoms. Lastly, although the current study participants were aware of the presence of their oral problems, it was not possible to determine whether treatment was necessary for those problems. However, in general, diseases with serious symptoms, such as toothaches, gingivitis, and chewing difficulties usually require treatment because they can significantly affect daily life and quality of life. Despite these limitations, this study has a strength in that it is the first social epidemiological study on marital status to use a large sample across Japan and focus on access to dental care.

## Conclusions

We found that the prevalence of non-dental treatment was significantly higher among non-married men than among married men even if they had oral symptoms. This study results indicate the importance of the implementation of a public dental health policy for protecting the health of non-married individuals.

## Data Availability

The datasets analyzed during the current study are not publicly available due to protect the participants’ identity but are available from the corresponding author on reasonable request with the permission of the Ministry of Health, Labour and Welfare.
